# High Prevalence, Genetic Diversity, and Recombination of *Porcine Sapelovirus* in Pig Farms in Fujian, Southern China

**DOI:** 10.3390/v15081751

**Published:** 2023-08-17

**Authors:** Qiu-Yong Chen, Zhi-Hua Sun, Yong-Liang Che, Ru-Jing Chen, Xue-Min Wu, Ren-Jie Wu, Long-Bai Wang, Lun-Jiang Zhou

**Affiliations:** 1 Institute of Animal Husbandry and Veterinary Medicine, Fujian Academy of Agriculture Sciences, Fujian Animal Disease Control Technology Development Center, Fuzhou 350013, China; fjchenqiuyong@163.com (Q.-Y.C.); cyl19760810@163.com (Y.-L.C.); fjchenrujing@163.com (R.-J.C.); wxm0593@163.com (X.-M.W.); wurenjie1231@163.com (R.-J.W.); 2 College of Animal Sciences (College of Bee Science), Fujian Agriculture and Forestry University, Fuzhou 350002, China; 18050768782@163.com

**Keywords:** *Porcine sapelovirus*, prevalence, diversity, recombination, Fujian Province

## Abstract

*Porcine sapelovirus* (PSV) is a ubiquitous virus in farmed pigs that is associated with SMEDI syndrome, polioencephalomyelitis, and diarrhea. However, there are few reports on the prevalence and molecular characterization of PSV in Fujian Province, Southern China. In this study, the prevalence of PSV and a poetical combinative strain PSV2020 were characterized using real-time PCR, sequencing, and bioinformatics analysis. As a result, an overall sample prevalence of 30.8% was detected in 260 fecal samples, and a farm prevalence of 76.7% was observed in 30 Fujian pig farms, from 2020 to 2022. Noteably, a high rate of PSV was found in sucking pigs. Bioinformatics analysis showed that the full-length genome of PSV2020 was 7550 bp, and the genetic evolution of its ORF region was closest to the G1 subgroup, which was isolated from Asia and America; the similarity of nucleotides and amino acids to other PSVs was 59.5~88.7% and 51.7~97.0%, respectively. However, VP1 genetic evolution analysis showed a distinct phylogenetic topology from the ORF region; PSV2020 VP1 was closer to the DIAPD5469-10 strain isolated from Italy than strains isolated from Asia and America, which comprise the G1 subgroup based on the ORF region. Amino acid discrepancy analysis illustrated that the PSV2020 VP1 gene inserted twelve additional nucleotides, corresponding to four additional amino acids (STAE) at positions 898–902 AAs. Moreover, a potential recombination signal was observed in the 2A coding region, near the 3′ end of VP1, owing to recombination analysis. Additionally, 3D genetic evolutionary analysis showed that all reference strains demonstrated, to some degree, regional conservation. These results suggested that PSV was highly prevalent in Fujian pig farms, and PSV2020, a PSV-1 genotype strain, showed gene diversity and recombination in evolutionary progress. This study also laid a scientific foundation for the investigation of PSV epidemiology, molecular genetic characteristics, and vaccine development.

## 1. Introduction

*Porcine sapelovirus* (PSV), taxonomically designated sapelovirus A, belongs to the genus *sapelovirus* within the family *Picornaviridae* [1]. PSV is a microsphere particle and single-positive-strand RNA virus, the smallest known animal RNA virus [2]. The average diameter of the PSV particle is about 28–30 nm, and the genome length is about 7.5 Kb, including a single open reading frame (ORF) encoding one leader protein (L), four structural proteins (VP4, VP3, VP2, VP1), and seven nonstructural proteins (2A, 2B, 2C, 3A, 3B, 3C, and 3D) [3]. Although PSV only has one serotype, it shows extensive genomic sequence diversity in different counties and districts [3,4,5,6,7]. The structure protein sequence near the 3′ end of the P1 region (VP4, VP3, VP2, and VP1) is a potential recombination hotspot, playing a pivotal role in the virus’ genetic diversity and evolution [8]. Traditionally, PSV has been thought to be composed of a single genotype PSV-1 [1]. However, Hungarian scholars recently discovered a potential novel genotype PSV-2 [9]; following this, some studies determined two genotype classifications by analyzing the genetic phylogenetic tree of PSV strains in the database [10].

PSV infections can cause either asymptomatic co-infection with other enteric pathogens or a symptomatic result in multisystemic wasting syndromes, such as encephalomyelitis, diarrhea, respiratory tract symptoms, and reproductive disorders of sows [11]. Experimentally, pregnant gilts infected with PSV developed fetal infections [12]. In an outbreak of PSV cases, 50~60-day-old piglets affected by PSV showed signs of acute diarrhea, respiratory disease, and polioencephalomyelitis [13]. The affected pigs, 3~4 weeks post-weaning, initially showed signs of either front or hind leg weakness, progressing to generalized weakness and lateral recumbency. The affected pigs maintained normal mentation throughout this period, and eventually died within a few days [14]. The gross lesions showed extensive congestion in small intestinal mucosa. In addition, PSV extra-intestinal tropisms appear as mild, non-suppurative myelitis, encephalitis, and pneumonia [13,14,15]. Histopathological examination (H&E) showed villous atrophy and crypt hyperplasia in the small intestine, hemorrhage in the lamina propria of the small intestine, and crypt fusion with epithelial cell hyperplasia in the large intestine [13,15]. The brain exhibited some characteristic neuronal vacuolization, perivascular cuffing, and severe lymphoplasmacytic and necrotizing polioencephalomyelitis with multifocal areas of gliosis and neuron satellitosis [15,16]. In addition, lung congestion, hyperemia, or hemorrhage were observed, and some alveoli ruptured to become large cysts [13,15]. Furthermore, immunohistochemistery (IHC) detected viral antigen co-localized in the spinal cord lesions [14] and epithelial cells of intestines [15] of affected pigs using an antibody known to react with PSV. Sapelovirus mRNA encoding VP1 protein was detected in neurons and nerve roots of the spinal cord using in situ hybridization (ISH) [16]. These laboratory observations of the diseased pigs in the outbreak are consistent with PSV being the causative agent.

PSV was first identified in England in 1960 and then reported in Japan, America, Republic of Korea, Italy, and other countries [7,16,17,18]. An epidemiological survey in China showed that the positive PSV detection rates in East China, Sichuan Province, and Hunan Province were 40.54%, 20.4%, and 46.39%, respectively, indicating that PSV is highly infectious in the pig population [5,19,20]. However, relevant study considering PSV have not been reported in Fujian Province, Southern China. In this study, we established a real-time PCR method and characterized the prevalence in Fujian pig farms. Moreover, a genome of PSV strain PSV2020 was identified, and the molecular characteristics were analyzed using bioinformatics. The present study aims to lay a foundation for the epidemiological investigation into and molecular genetic characterization of PSV in Fujian Province.

## 2. Materials and Methods

### 2.1. Samples, Strain, and Materials

For the epidemiological survey of PSV, a total of 260 clinical samples, including feces, fecal swabs, and intestine samples, were collected between 2020 and 2022 from sows and suckling, post-weaning, and fattening pigs that were asymptomatic or with clinical diarrhea (25 with clinical diarrhea and 5 asymptomatic) from 30 farms in Fujian Province. A description of the studied pig population and the number of collected samples for each farm is shown in Supplementary Table S1. All samples were detected for PSV using real-time PCR. The PSV2020 strain was preserved by the Institute of Animal Husbandry and Veterinary Medicine, Fujian Academy of Agricultural Sciences. M-MuLV First Strand cDNA Synthesis Kit (Code No: B532435), Rapid Competent Cell Preps Kit (One-step, Code No: B529307), SanPrep Column Plasmid Mini-Preps Kit (Code No: B518191), SanPrep Spin Column & Collection Kit (Code No: B515103), and Taq Plus DNA polymerase, 2 × Taqman Fast qPCR Mix (High Rox) (Code No: B639276, BBI) were bought from Sangon Biotech (Shanghai, China). RNA extraction kit (Code No: 9767), PrimeScriptTM RT Master Mix Reverse Transcription Kit (Code No: RR036A), TaKaRa LA Taq^®^ (Code No: RR02MQ), and pMD 18-T Vector (Code No: 6011) were purchased from TaKaRa Bio Inc. (Daliang, China).

### 2.2. Primer Design and Synthesis

Since the 5′ UTR gene region of PSV is highly conserved, it was selected as the molecular target for the real-time PCR. The real-time primer sequences are shown in Table 1. The V13 strain (GenBank ID: NC_003978.1) genome was considered as the reference sequence to design ten pairs of primers for amplification of the PSV genome internal sequence, six specific primers, and four universal adapter primers for 5′ RACE and 3′ RACE experiments. All full-length genome amplification primers were shown in Supplementary Table S2. The primers and probe were synthesized by Sangon Biology (Shanghai) Co., Ltd. (Shanghai, China).

### 2.3. Preparation of Standard Plasmid

Aliquots of 0.5 g of fecal material were re-suspended in 1 mL of ice-cold phosphate-buffered saline (PBS), vortexed for 1 min, and clarified using centrifugation at 12,000× *g* for 5 min, and the PSV total RNA (0.2 mL of the supernatant of samples) was isolated using the RNA extraction kit. Then, reverse transcription from RNA to cDNA was performed using the RT kit, stored at −80 °C. Utilizing the cDNA as template and PSV-F and PSV-R as specific primers, amplification was carried out according to the 25 µL reaction system, including Taq Plus DNA Polymerase (5 U/µL), 0.5 µL; 10× PCR Buffer, 2 µL; PSV-F, 0.5 µL; PSV-R, 0.5 µL; dNTP (10 mM), 0.5 µL; MgCl_2_ (25 mM), 0.5 µL; cDNA, 2 µL; and ddH_2_O, 17 µL. Reaction conditions of 95 °C for 3 min; 95 °C for 30 s, 57 °C for 30 s, 72 °C for 30 s, 35 cycles, and 72 °C for 8 min were used for full-length elongation. PCR amplificons were analyzed using 1% agarose gel electrophoresis. Target fragments were recovered using a SanPrep kit, then ligated into pMD^®^ 18-T Vector, and recombinant plasmids were transformed into DH-5α competent cells. The ampicillin plate was used for screening the positive recombinant plasmid, and a single colony was selected for the next culture. The positive recombinant plasmid (PSV-T) was obtained by using the SanPrep column plasmid DNA mini extraction kit and was identified via PCR and Sanger sequencing. The standard plasmid was quantified using a NanoDrop2000 spectrophotometer (Thermo Fisher Scientific, Waltham, MA, USA), and copy numbers of PSV-T were calculated following the method described by Lee et al. [21] then diluted tenfold (4.67~4.67 × 10^7^ copies/µL) and stored at −80 °C.

### 2.4. Establishment of qPCR Method and Standard Curve

To obtain the lowest threshold cycle (Ct) and maximum fluorescence intensity, the concentration of the primers and probe and the annealing temperature were optimized. The best parameters were selected after all the reactions had been conducted in triplicate. The optimized real-time PCR system included. 2 × TaqMan Fast qPCR master mix, 10 µL; PSV-F, 0.4 µL; PSV-R, 0.4 µL; PSV-Taq, 0.4 µL; ddH_2_O, 7.8 µL; and standard plasmid (PSV-T), 1 µL. The reaction conditions were 95 °C for 3 min; 95 °C for 10 s, 57 °C for 15 s, and 72 °C for 30 s. The optimized real-time PCR detection method was used to amplify the recombinant plasmid with a concentration of 4.67 × 10^1^~10^7^ copies/µL, repeated three times in succession to obtain the amplification curve and standard curve. The standard logarithm (standard curve) was derived with the common logarithm (lgC) of the standard starting copy number as the abscissa and the cycle threshold (Ct value) as the ordinate.

### 2.5. Specificity, Sensitivity, and Repeatability Test

The DNA or cDNA of CSFV, PRV, PRRSV, PEDV, TGEV, PoRV, PDCoV, and PTV were used as templates for evaluating the specificity of the established real-time PCR. When the curve was above the threshold before 35 cycles had passed, we judged the result to be positive. The detection limit of the established real-time PCR was carried out using tenfold dilution plasmids with a concentration of 4.67~4.67 × 10^7^ copies/µL; each dilution was amplified via real-time PCR with three replicates and independent experiments carried out three times. The repeatability test was carried out with 4.67 × 10^2^, 4.67 × 10^4^, and 4.67 × 10^6^ copies/µL plasmids, repeated three times. The intra- and inter-assay of the method were determined by calculating the coefficient of variation (CV).

### 2.6. Full-Length Genome Amplification, Sequencing, and Annotation of PSV

Reverse transcription (RT) was performed using a PrimeScriptTM RT Master Mix reverse transcription kit according to the manufacturer’s recommendations and a primer 3′ adapter. The viral RNA covering the internal genome was amplified via RT-PCR with primers based on the nucleotide sequences of the V13 strain (GenBank ID: NC_003978.1) (Supplementary Table S1), according to the instructions and using a 25 µL reaction system: 2 × GC Buffer I, 12.5 µL; forward (No: 1~10) and reverse (No: 1~10) primers of 0.5 µL, respectively; dNTP (2.5 mM), 4 µL; ddH_2_O, 6.3 µL; cDNA, 1 µL; and Taq polymerase (5 U/µL), 0.2 µL. The reaction conditions were 95 °C for 3 min; 94 °C for 10 s, 55 °C for 15 s, 72 °C for 30 s, 35 cycles, and 72 °C for 7 min. The 3′ RACE experiments included two steps. Firstly, reverse transcription of the RNA into cDNA with 3′ adaptor primer was carried out according to the manufacturer’s instructions, and then nested PCR was performed using 3′ RACE-F1 and 5-3′ outer, or 3′ RACE-F2 and 5-3′ inner, as primers (Supplementary Table S1). 5′ RACE includes three steps. First-strand cDNA synthesis was primed using specific primers 5′ RACE-RT1 and 5′ RACE-RT2 (Supplementary Table S1). Then, a homopolymeric tail was added to the 3′-end of the cDNA after being treated with RNase H and TdT. Thirdly, nested PCR amplification was accomplished using Taq DNA polymerase, 5′ adaptor primer and 5′ RACE-R1, or 5-3′ outer and 5′ RACE-R2 (Supplementary Table S1) according to the Invitrogen 5′ RACE system manual. The PCR amplificons were tested using 1% agarose gel electrophoresis, and the target segments were recovered. The purified DNA was ligated into the pMD18-T vector and transformed into DH-5α competent cells, using an ampicillin plate for screening, and a single colony was selected for culturing. The recombinant plasmids were isolated by using the SanPrep column plasmid DNA mini extraction kit and identified using PCR and Sanger sequencing. The above 12 fragments were sent to Sangon Biotech (Shanghai) for Sanger sequencing and assembled using the SeqBuilder program (DNAStar, Madison, WI, USA) to obtain the PSV2020 strain whole-genome sequence. Referring to the V13 strain, the PSV2020 genome was annotated with protein coding, and the genome structure map was drawn with IBS 2.0 software http://ibs.biocuckoo.org/ (accessed on 26 April 2023).

### 2.7. PSV2020 Genome Homology, Genetic Evolution, and Recombination Analysis

Bioinformatics analyses were performed with 61 strains obtained from the GenBank database (Supplementary Table S3). The nucleic acid and amino acid homology of the complete open reading frame (ORF) between the PSV2020 strain and reference strains were analyzed using MegAlign software (DNAStar, Madison, WI, USA), and the phylogenetic analyses of the complete ORF, VP1, and 3D nucleotide sequences were performed using Mega 7.0 software (Tempe, AS, USA). RDP 4.0.1 (Cape Town, WC, South Africa) and Simplot 3.5.1 software (Baltimore, MD, USA) were used to test potential recombination events of the PSV2020 strain.

## 3. Results

### 3.1. Standard Curve

Optimized TaqMan-based real-time PCR was used to amplify standard plasmids with a dilution concentration of 4.67~4.67 × 10^7^ copies/µL (Supplementary Figure S1A), and the logarithm of the plasmid copy number was plotted against the corresponding Ct values to construct the standard curve (Supplementary Figure S1B). The initial template DNA concentration showed a linear correlation with the Ct value. The correlation coefficient R^2^ value was 0.999, the slope was −3.107, and the intercept was 39.94. That is, the established standard curve was y = −3.107x + 39.94.

### 3.2. Specificity, Sensitivity, and Repeatability of PCR Assay

The specificity test results showed that the method did not show signals for other pathogens (including CSFV, PRV, PRRSV, PEDV, TGEV, PoRV, PDCoV, and PTV) but only showed a significantly amplified signal of PSV (Supplementary Figure S1C), indicating that the established real-time PCR method was highly specific for PSV identification. From the kinetic curve of real-time PCR, the real-time PCR limit of detection was 46.7 copies/µL (Supplementary Figure S1A), and the sensitivity was 10 times higher than conventional PCR (Supplementary Figure S1A,D). For the repeatability results of the real-time PCR method, the coefficient of variation (CV) of intra-assay was 0.14~0.47% and of inter-assay was 0.76~0.83%; all coefficients were less than 1% (Table 2).

### 3.3. The Prevalence of PSV in Pig Farms in Fujian Province

PSV was detected in 260 samples across all 30 farms. Globally, 23 out of 30 (76.67%) farms were positive, and the rate of positive samples was 30.8% (80/260). However, different age distribution patterns were observed regarding the positive rates. Remarkably, a 60.0% farm prevalence and 41.18% sample prevalence were observed in suckling pigs, which were higher than those in sows, post-weaning pigs, and fattening pigs (Table 3). It is noteworthy that sows had a significantly higher farm prevalence (56.0%) than post-weaning pigs (35.71%) and fattening pigs (22.22%). Positive samples for PSV had CT values of 18.90~34.72 (Supplementary Table S4), corresponding to an amount of PSV RNA ranging from 4.78 × 10^4^ to 5.89 × 10^9^ genomic copies per mL of supernatant. Notably, high PSV viral loads (6.75 ± 0.85, log10 copies/mL) were also identified in sows.

### 3.4. Whole-Genome Amplification, Sequencing, and Annotation of PSV2020

The PCR amplicons were analyzed using 1% agarose gel electrophoresis, showing that we amplified the 10 target fragments (Supplementary Figure S2A), covering the internal sequence of the virus genome (234~7302 nt). Through RACE technology, we amplified the 3′ end (Supplementary Figure S2B) and 5′ end of the PSV genome (Supplementary Figure S2C). The above 12 fragments were sequenced for assembling the full-length genome of PSV. The genome sequence of PSV2020 was submitted to GenBank under accession ON146286.1. The amino acid sequences were then deduced via alignment with the amino acid sequence of the V13 strain (GenBank ID: NC_003978.1). The sequence analyses revealed that the genomes of PSV2020 consisted of 7550 nucleotides. The 5′-terminal untranslated region (5′ UTR) and 3′ UTR contained 446 and 96 nucleotides and contained a single large open reading frame (ORF) with 7008 nucleotides encoding 2336 amino acids (Table 4). The genome organization of PSV2020 was considered to be similar to that of known PSV strains, and the polyprotein was considered to be cleaved into twelve mature peptides consisting of an L protein; the four structural proteins VP4, VP2, VP3, and VP1; and the seven non-structural proteins 2A, 2B, 2C, 3A, 3B, 3C, and 3D.

### 3.5. Phylogenetic Analyses of Complete ORF, VP1, and 3D Gene

**Figure 1:**
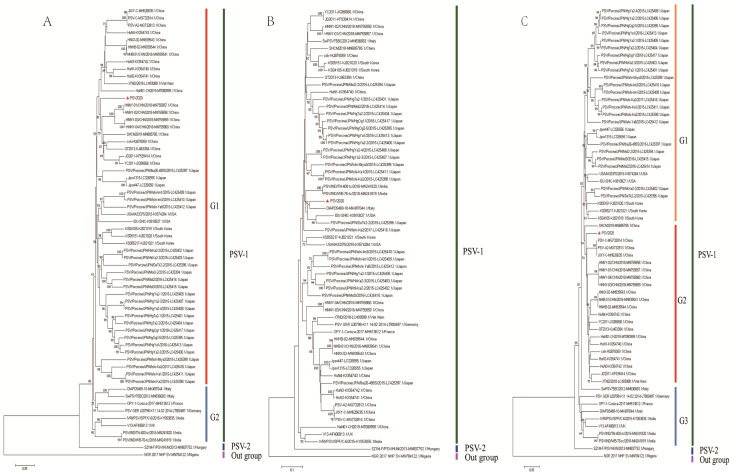
Phylogenetic analysis of PSV2020 complete ORF, VP1, and 3D genes. (**A**) Phylogenetic tree based on the ORF nucleic acid sequence. (**B**) Phylogenetic tree based on the VP1 gene nucleic acid sequence. (**C**) Phylogenetic tree based on the 3D gene nucleic acid sequence. The trees were constructed using the neighbor-joining method. The percentages of replicate trees in which the associated taxa clustered together in the bootstrap test (1000 replicates) are shown next to the branches (>50%). The red triangle represents the PSV2020 isolated in this study.

A phylogenetic tree was constructed based on the alignment of complete ORF nucleotide sequences using data on PSV2020 and other PSVs retrieved from GenBank (Supplementary Table S3). PSVs were phylogenetically divided into three clades (PSV-1, PSV-2, and outgroup subgroups), wherein the PSV-1 clade could be classified into G1 and G2 subgroups; the G1 subgroup is composed of Asian (China, Japan, Republic of Korea, and Viet Nam) and American (USA) isolated strains, whilst the G2 subgroup consisted of strains detected in Europe (Italy, France, Germany, and the UK) and India. The PSV-2 subgroup was made up of the new genotype SZ1M-F/PSV from Hungary, while the monkey strain NGR2017NHP-SV belongs to the outgroup cluster. The PSV2020 strain is grouped into the G1 subgroup (Figure 1A). Phylogenetic analyses of the VP1 and 3D nucleotide sequences were also conducted. In the phylogenetic tree based on the VP1 gene, the PSV2020 strain was divided into three clades (Figure 1B), in the same manner as the tree of complete ORF, and the PSV-1 clade could be divided into G1, G2, and G3 subgroups. The PSV2020 strain was grouped into the G1 subgroup (Figure 1B), but it was different in terms of the genetic evolution of the ORF region, which was far from the G1 subgroup strains of ORF (Figure 1A). However, it was closest to the strains detected in Italy, America, and Japan (Figure 1B). A 3D-based tree showed that PSVs were phylogenetically separated into three different groups and formed G1, G2, and G3 subgroups in the PSV-1 group. PSV2020 had the closest genetic relationship with the G2 subgroup which comprised Chinese and Vietnamese isolated strains, and it was distinct from the G1 subgroup composed of Japanese, Korean, and American isolates and the G3 subgroup composed of European isolates (Figure 1C). These results suggest that the genes of PSV2020 are diversifying and undergoing the process of genetic evolution.

### 3.6. Homology Analyses of Complete ORF

**Figure 2:**
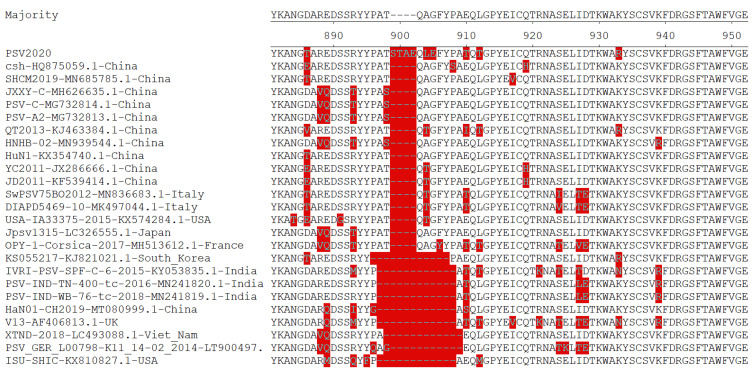
Alignment of the amino acid sequences near the 3′ end of the VP1 gene among PSV2020 and other PSVs. Sequences in red show the hypervariable region in the C-terminus of VP1, and four additional AAs (STAE) inserted between 898 and 902 of the complete ORF as compared with all other known PSVs.

Homology comparison of the complete ORF of PSV2020 with those of other PSV strains retrieved from GenBank (Supplementary Table S3) revealed nucleotide and amino acid identities ranging from 59.5% to 88.7% and from 51.7% to 97.0%, respectively (Table 5). Among PSV-1 group strains, the PSV2020 strain nucleotide and amino acid identities demonstrated the highest homology in the G1-Asia subgroup, 84.9~88.7% and 93.7~97.0%, respectively, and the lowest homology in the G2-Europe subgroup, 84.1~85.9% and 93.2~96.2%, respectively. In addition, PSV2020 had sequence identities of 77.3% (nucleotides) and 84.3% (amino acid) with the PSV-2 strain, a potentially novel genotype of PSV in Hungary [9]. The nucleotide and amino acid homology between PSV2020 and the outgroup formed by the NGR2017NHP-SV strain detected in monkeys was the lowest with 59.5% (nucleotides) and 51.7% (amino acid), respectively. Moreover, the amino acid homology analysis of VP1 showed that the PSV2020 strain contained twelve additional nucleotides, corresponding to four additional AAs (STAE) inserted at positions 898–902 AAs of the complete ORF compared with other reference PSV strains (Figure 2).

### 3.7. Recombination Analyses of PSV2020

**Figure 3:**
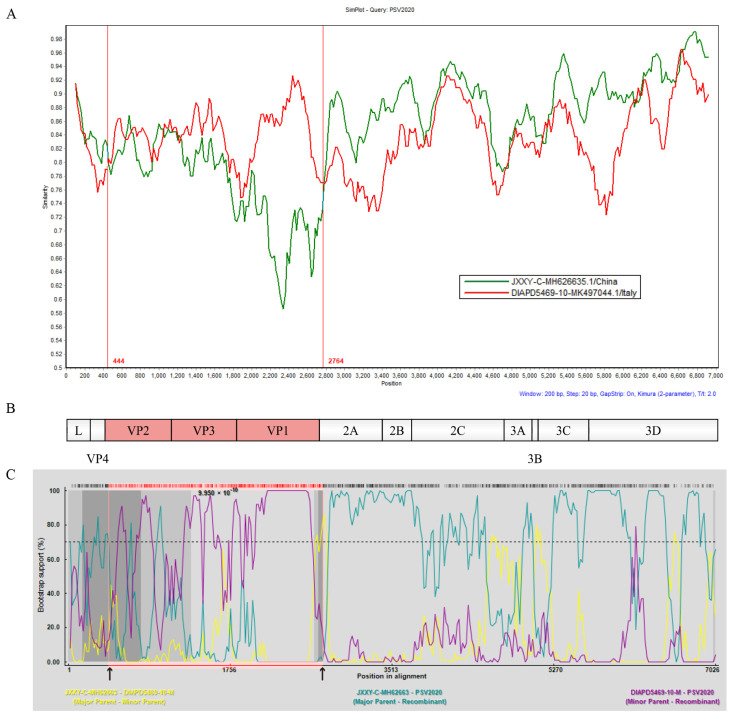
Identification of the recombination events was based on the complete ORF of the potential recombinants. (**A**) Similarity analysis of the complete ORF of the potential recombinants using the SimPlot 3.5.1 program with a 200 bp window, 20 bp step size, and 100 bootstrap replicates. PSV2020 represents the query sequence, and DIAPD5469-10 (red line) and JXXY-C (green line) represent the potential parental strains. The vertical line (red line) is the potential breakpoint. (**B**) Genome origination of PSV2020 based on the complete ORF using IBS2.0. The red area indicates the region of origin from DIAPD5469-10, and the white area indicates the region of origin from JXXY-C. (**C**) Recombination breakpoint analyses were conducted using RDP 4.0.1 with the sequences of PSV2020, DIAPD5469-1, and JXXY-C. The recombination in PSV2020 predicted by the analyses using the BootScan method is shown. The cut-off value in the bootstrapping test (>70%) is indicated by a dotted line, and the black arrows indicate putative recombination breakpoints.

To obtain evidence of genetic recombination in PSV2020, recombination analyses were conducted using SimPlot 3.5.1 software and RDP 4.0.1. Standard similarity plot analysis illustrated that the sequences of VP2, VP3, and VP1 genes of PSV2020 were highly similar to the Italian strain DIAPD5469-10, whereas the 2A~3D gene region was highly similar to the Chinese strain JXXY-C (Figure 3A,B), suggesting one recombination event in its evolutionary history; DIAPD5469-10 and JXXY-C represent potential parental strains, respectively. The recombination breakpoint analyses of PSV2020 were performed using seven different methods: RDP, Chimaera, BootScan, 3Seq, GENECONV, MaxChi, SiScan. BootScan showed potential recombination breakpoints located in the VP2 and 2A regions (*p* = 9.950 × 10^−10^) (Figure 3C), indicating that PSV2020 had G2-like capsid proteins (VP2, VP3, and VP1) in the genetic backbone of G1 viruses (G1 and G2 subgroups based on ORF, Figure 1A). These findings suggested that PSV2020 might have evolved from an ancestor that emerged through genetic recombination between G1 and G2 viruses.

## 4. Discussion

Although *porcine sapelovirus* is a ubiquitous virus in farmed pigs and wild pigs, infections have been associated with a wide spectrum of symptoms. In China, the prevalence of PSV has been shown to range from 20.4% to 46.39% in different provinces, and an outbreak of gastroenteritis associated with respiratory disease and polioencephalomyelitis was reported in Shanghai [13]. In the UK, the disease occurred in pigs 3~4 weeks post-weaning in 2008 involving approximately 35 cases; the affected animals initially showed signs of nervous disease, either front or hind leg weakness progressing to generalized weakness, and then died within a few days [14]. In the USA, approximately 3000 11-week-old finishing pigs experienced an outbreak of PSV disease with an overall morbidity of 20% and a case fatality rate of 30%. Moreover, sows experimentally injected with PSV via the intrauterine route on day 30 of gestation resulted in a fetal mortality rate of 94.4% [16]. PSV infections were also reported in Japan, Republic of Korea, France, Zambia, Vietnam, Italy, Brazil, and the Czech Republic [3,8,17,18,22,23,24,25]. Notably, an epidemiological investigation revealed that the PSV-positive rates and co-infection of other enteric pathogens in diarrhea samples were much higher than in asymptomatic samples, indicating that PSV may contribute collectively to the enteric disease of pigs along with other porcine pathogens [7,19,24,25]. Therefore, PSV cannot be ignored when controlling diarrhea in pigs, and establishing an efficient and specific diagnostic method is thus a matter of urgency. To investigate the epidemic of PSV, we established a TaqMan-based real-time PCR diagnostic method based on the conserved region of PSV 5′UTR. The present method demonstrates high specificity and the best batch repeatability between groups and within groups. The sensitivity is 10 times higher than that of traditional PCR methods and one order of magnitude higher than that of diagnostic methods established by other researchers [26,27], with a minimum of 46.7 copies/µL. We also demonstrated that the Fujian pig farms were highly infected with PSV (76.67%) (Table 3). Additionally, a particularly high infection rate was found in suckling pigs (41.18%), sows (25%), and post-weaning pigs (26.8%) compared with fattening pigs (16.7%). In previous reports, a higher PSV prevalence (30.36%) was found in nursery pigs than in suckling pigs (0%) in Jiangxi Province, China [27], and fattening pigs had a significantly higher PSV prevalence (94.0%) than suckling pigs (36.2%) in Zambia [3]. PSV was identified in 13.16% (15/114) of the suckling pigs, 60.15% (80/133) of the nursery pigs, and 69.41% (59/85) of the fattening pigs in Hunan Province, China [5]. The age distribution patterns regarding the PSV prevalence in this study differed from previous reports. This may be because PSV can coexist with sows over the long term when asymptomatic, then sows become PSV carriers and PSV is transmitted horizontally from the sows to the suckling pigs during nursing. Thus, more research is needed to explain the high PSV infection rate in sows.

The PSV genome contains only one open reading frame (ORF), which encodes a polyprotein precursor, which is further cleaved into 12 mature functional proteins by self-encoded proteases [7,22]. Through amplification, sequencing, and prediction, a genome of the PSV strain PSV2020 was identified, and the molecular characteristics were analyzed using bioinformatics. The ORF region of the PSV2020 strain is 7008 bp and encodes 2336 amino acids (AAs). The nucleotide and amino acid homology with other PSVs are 51.7~88.7% and 51.7~97.0%, respectively (Table 5). Differences in the ORF sequences were significantly related to the insertions of AAs occurring in the C-terminal region of VP1; PSV2020 contained twelve additional nucleotides, corresponding to four additional AAs (STAE) inserted at positions 898–902 AAs of the complete ORF compared with other reference PSV strains (Figure 2). The C-terminus of the VP1-VP3 proteins located on the surface of the virion significantly affects the antigenicity of PSV and is the main position that induces the host to produce neutralizing antibody [28,29]. Therefore, this may be related to the escape of the host immune response to PSV in the process of genetic evolution and may explain the high rates of PSV infection in pigs, and it may also be a molecular marker of potential epidemic strains of PSV [27,30].

Phylogenetic analysis based on gene sequencing is widely used to determine the taxonomy of picornaviruses [18,31,32]. Phylogenetic analysis based on PSV ORF sequences indicates that PSV2020 with a closely related strain from the Asian and American strains fell into the PSV-1 genotype G1 subgroup and far from the PSV-2 genotype reported in Hungary (Figure 1A). However, phylogenetic analysis of VP1 gene sequences of PSV2020 showed a distinct phylogenetic topology with those of ORF sequences (Figure 1B), and closer to the DIAPD5496-10 strain reported in Italy than the Asian and American strains. Additionally, 3D protein is the specific RNA polymerase of the virus and has the ability to synthesize RNA. It is a pivotal protein that mediates the replication ability of the virus [33]. The phylogenetic tree constructed based on the 3D sequence shows that PSV2020 is closest to the Chinese isolated strain (Figure 1C), and all PSVs showed that the 3D gene has certain regional genetic conservation that is relatively conservative in the evolution process. These results suggest that PSV2020 shuffled on the phylogenetic trees for these different genomic regions and has genetic diversity in the epidemic process, indicating that the ORF, VP1, and 3D regions evolved independently. Viral genetic variation and recombination are the main genetic evolution modes of picornaviruses and play an important role in the incongruent tree topologies [23,34,35]. Therefore, we suspect that recombination events occur in PSV2020.

In this study, similarity plot analysis showed that the structural proteins (VP2, VP3, and VP1) of the PSV2020 P1 region were highly similar to the DIAPD5469-10 strain, which was representative of the G2 European subpopulation (Figure 3A), and the recombination signal occurred at 444 and 2764 AA, while the P2 (2A, 2B, and 2C) and P3 (3A, 3B, 3C and 3D) regions were highly similar to the Chinese strain JXXY-C, which was representative of G1 subgroup (Figure 3A,B). BootScan analysis demonstrated potential breakpoints located in the VP2 and 2A regions (Figure 3C), which were consistent with the similarity plot analysis. These analyses indicate that PSV2020 is a recombination virus. Recently, some recombination events among PSVs have been reported and the possible recombinant hotspots are predicted to be located in an upstream region of the 2B gene, near the 3′ end of the P1 region, or in the L and 2A regions [3,4,5]; nevertheless, the PSV2020 recombination hotspots located in the VP2 and 2A regions indicate that this is not completely consistent with previous reports. Thus, it might be possible that PSVs acquire higher pathogenicity through several genetic recombination events that result in some outbreaks of various severe illnesses caused by PSV infection [14,16]. Currently, only a few PSVs have been detected from recombination events due to a limited complete sequence, and more circulating PSVs are expected to be acquired and completely sequenced to clarify the recombination rules.

## 5. Conclusions

In conclusion, we have established strong specificity, high sensitivity, and good repeatability from TaqMan-based real-time PCR, indicating that an overall sample prevalence of 30.8% was detected in 260 fecal samples, and a farm prevalence of 76.7% was observed in 30 Fujian pig farms, collected from 2020 to 2022. Meanwhile, a PSV-1 genotype strain, PSV2020, showed gene diversity and recombination events while undergoing genetic evolution. These results also lay a scientific foundation for the investigation of PSV epidemiology, molecular genetic characteristics, and vaccine development.

## Figures and Tables

**Table 1:** The primers for real-time fluorescence quantitative PCR.

Primer	Sequences (5′-3′)	Product Size (bp)
PSV-F	GATACACTTAAATGGCAGTAGCGT	129
PSV-R	CTCACTGTCTACTCTCCTGTAACCA	
PSV-taq	FAM-CAATTGTCGATAGCCAT-MGB	

**Table 2:** Intra- and inter-assay variability analysis of the real-time PCR assay.

Plasmid	Concentration (Copies/µL)	Intra-Assay		Inter-Assay	
		$\mathrm{Mean}\;(\bar{X}\pm \mathrm{SD})$	CV (%)	$\mathrm{Mean}\;(\bar{X}\pm \mathrm{SD})$	CV (%)
PSV-P	4.67 × 10^2^	31.53 ± 0.15	0.47	31.26 ± 0.26	0.83
	4.67 ×10^4^	25.23 ± 0.03	0.14	25.10 ± 0.19	0.76
	4.67 × 10^6^	19.41 ± 0.07	0.36	19.47 ± 0.15	0.78

**Table 3:** Detection results of PSV in the clinical samples collected from 2020 to 2022 in pig farms in Fujian Province using real-time PCR.

Age Group	No. of Farms	Farm Prevalence	No. of Samples	Sample Prevalence	$\mathrm{Concentration}\;(\bar{X}\pm \mathrm{SD}$, log10 Copies/mL)
Suckling pigs (0–28 days)	25	60.0% (15/25)	102	41.18% (42/102)	6.99 ± 0.80
Post-weaning pigs (29–60 days)	28	35.71% (10/28)	56	26.8% (15/56)	7.05 ± 1.90
Fattening pigs (61 days–25 weeks)	18	22.22% (4/18)	30	16.6% (5/30)	6.03 ± 0.39
Sow	30	56.0% (14/30)	72	25.0% (18/72)	6.75 ± 0.85
Total	30	76.67% (23/30)	260	30.8% (80/260)	

**Table 4:** The length of each gene in PSV2020 strains.

Number	Gene	Location	Length
1	5′UTR	1–446	446
2	L	447–698	252
3	VP4	699–857	159
4	VP2	858–1571	714
5	VP3	1572–2273	702
6	VP1	2274–3164	891
7	2A	3165–3842	678
8	2B	3843–4157	315
9	2C	4158–5153	996
10	3A	5154–5453	300
11	3B	5454–5519	66
12	3C	5520–6065	546
13	3D	6066–7454	1389
14	3′UTR	7455–7550	96

**Table 5:** The homology analysis of PSV2020 ORF nucleotides and amino acids.

PSV2020 VS	PSV-1				PSV-2	Out Group
	G1-Asia	G1-America	G2-Europe	G2-India		
ORF nucleotides	84.9–88.7%	84.6–86.0%	84.1–85.9%	84.2–85.7%	77.3%	59.5%
ORF amino acids	93.7–97.0%	94.2–95.4%	93.2–96.2%	93.8–95.6%	84.3%	51.7%

## Data Availability

All data are reported in this article.

## Supplementary Materials

The following supporting information can be downloaded at: https://www.mdpi.com/article/10.3390/v15081751/s1, Table S1: Description of the studied population; Table S2: The primers for PSV whole-genome amplification; Table S3: List of all PSVs used in this study; Table S4: The original detect results of PSV in the clinical samples in Fujian pig farms via qPCR; Figure S1: Establishment of TaqMan-based real-time PCR for PSV; Figure S2: PSV whole-genome PCR amplicon gel electrophoresis.
