# Supplementary material for: High Prevalence, Genetic Diversity, and Recombination of Porcine Sapelovirus in Pig Farms in Fujian, Southern China
Source: Viruses. 2023 Aug 17;15(8):1751. doi: 10.3390/v15081751 (PMC10458035; doi:10.3390/v15081751)
Supplement: Supplementary file 1 [file viruses-15-01751-s001.zip › Supplementary material -Table/Supplementary_Material-Table S2 The primers for PSV whole genome amplification.pdf]

Supplementary Table S2 The primers for PSV whole genome amplification

No. of Primer	Primer	Sequence (5'-3')	Product size (bp)
1	PSV-F234	AGAGTAGACAGTGAGCTATGGGCA	808
	PSV-R1042	TAAAACCTATCTACAGATGTACCAGGAGT	
2	PSV-F922	TAACAACACAAGAGGCAGCCA	401
	PSV-R1323	GCTCTGTTGGATAGTTGTTACCCT	
3	PSV-F1181	TGTACAGTGTAATGCATCTAAATTCCA	722
	PSV-R1903	ACAACAGATCCTCTGTAATTTGCATA	
4	PSV-F1788	ACACAAGGTAACCAGACAGCCAG	784
	PSV-R2572	AGATGTCTCCTACCACTTGCGTT	
5	PSV-F2414	TGAATCTGACACTAACTCTGGTGGA	813
	PSV-R3227	TGTAAATGAACCTCTATCAAACCTAACT	
6	PSV-F3068	TAAAGCTAATGGTGATGCCAGACA	958
	PSV-R4026	CCAAAGTGGACATTTTACACTCATCT	
7	PSV-F3832	TGTCTGGAGAAGTTAAGAAACAAGTG	969
	PSV-R4801	GGAGGCCAACACAAAATCAGAA	
8	PSV-F4578	GATAATCAGAGACCCTATACCCTTCC	1003
	PSV-R5581	TGTTATCATAAATGCCCAATCCAG	
9	PSV-F5390	GGTTAGACTGTTTTCCACAAAGCA	1015
	PSV-R6405	CTTTCTTTCTAATCCCCATGGTGT	
10	PSV-F6256	TGGTTTCCAAATTTAAGGCAATAG	1046
	PSV-R7302	CACCATTGTGCCATGCCA	
11	3' RACE-F1	TTGGTTGCCTATCCCTATGAATTGGATC	
12	3' RACE-F2	CAGCAGACAAATCAACAACTTTCCAAACAG	
13	5' RACE-R1	TTCCATGGGGGGGTTTCAACTGACTA	
14	5' RACE-R2	GTATAGGCCCAGTCCAGGTTTCCCAA	
15	5' RACE-RT1	GGTTTAGTGAACTGAGAGGGA	
16	5' RACE-RT2	GTTCCATGCCTGAGAATAGT	
17	5' adaptor	GCTGTCAACGATACGCTACGTAACGGCATGACAGTG CCCCCCCCCCCCCCC	
18	3' adaptor	GCTGTCAACGATACGCTACGTAACGGCATGACAGTG TTTTTTTTTTTTTTTTT	
19	5-3' outer	GCTGTCAACGATACGCTACGTAAC	
20	5-3' inner	GCTACGTAACGGCATGACAGTG	
