# Supplementary material for: High Prevalence, Genetic Diversity, and Recombination of Porcine Sapelovirus in Pig Farms in Fujian, Southern China
Source: Viruses. 2023 Aug 17;15(8):1751. doi: 10.3390/v15081751 (PMC10458035; doi:10.3390/v15081751)
Supplement: Supplementary file 1 [file viruses-15-01751-s001.zip › Supplementary material -Table/Supplementary_Material-Table S3 List of all PSVs used in this study.pdf]

Supplementary Table S3 List of all PSVs used in this study

NO	Virus isolate	Collection date	Genbank Accession No.	Geographic origin	Sequence length
1	csh	2009	HQ875059.1	China	7502
2	KS055217	2005	KJ821021.1	South Korea	7542
3	KS05151	2005	KJ821020.1	South Korea	7566
4	KS04105	2004	KJ821019.1	South Korea	7542
5	YC2011	2011	JX286666.1	China	7572
6	JD2011	2011	KF539414.1	China	7572
7	IVRI/PSV/SPF/C-6/2015	2015	KY053835.1	India	7491
8	PSV/IND/TN-400-tc/2016	2016	MN241820.1	India	7467
9	PSV/IND/WB-76-tc/2018	2018	MN241819.1	India	7494
10	HaN01-CH2019	2019	MT080999.1	China	7551
11	SwPSV75BO2012	2012	MN836683.1	Italy	7560
12	SHCM2019	2019	MN685785.1	China	7567
13	JXXY-C	2017	MH626635.1	China	7557
14	DIAPD5469-10	2015	MK497044.1	Italy	7564
15	PSV-C	2016	MG732814.1	China	7564
16	PSV-A2	2016	MG732813.1	China	7564
17	USA/IA33375/2015	2015	KX574284.1	USA	7565
18	V13	UK	AF406813.1	Germany	7491
19	HNNY-04/CHN/2018	2018	MN755860.1	China	7576
20	HNNY-03/CHN/2018	2018	MN755859.1	China	7576
21	HNNY-02/CHN/2018	2018	MN755858.1	China	7576
22	HNNY-01/CHN/2018	2018	MN755857.1	China	7576
23	SZ1M-F/PSV/HUN/2013	2013	MN807752.1	Hungary	7534
24	NGR_2017_NHP_SV	2017	MN784122.1	South Korea	8207
25	Jpsv447	2009	LC326556.1	Japan	7582
26	Jpsv1315	2009	LC326555.1	Japan	7569
27	XTND/2018	2018	LC493088.1	Viet Nam	7537
28	PSV_GER_L00798-K11_14-02_2014	2018	LT900497.1	Germany	7499
29	HNHB-02	2016	MN939544.1	China	7523
30	HNXX-02	2017	MN939543.1	China	7517
31	HNHB-01/CHN/2016	2016	MN939541.1	China	7518
32	OPY-1-Corsica-2017	2017	MH513612.1	France	7532
33	ISU-SHIC	2016	KX810827.1	USA	7389
34	HuN4	2015	KX354743.1	China	6,999
35	HuN3	2015	KX354742.1	China	6996
36	HuN2	2015	KX354741.1	China	6996
37	HuN1	2015	KX354740.1	China	6996
38	QT2013	2013	KJ463384.1	China	7532
39	PSV/Porcine/JPN/Ishi-Ka2/2017	2017	LC425416.1	Japan	7534
40	PSV/Porcine/JPN/MoI3/2016	2016	LC425415.1	Japan	7540
41	PSV/Porcine/JPN/HgOg11/2018	2018	LC425417.1	Japan	7521
42	PSV/Porcine/JPN/MoI2/2016	2016	LC425414.1	Japan	7514
43	PSV/Porcine/JPN/HgYa1/2016	2016	LC425413.1	Japan	7526
44	PSV/Porcine/JPN/Ishi-Ya8/2015	2015	LC425412.1	Japan	7528
45	PSV/Porcine/JPN/Ishi-Ka1/2015	2015	LC425411.1	Japan	7545
46	PSV/Porcine/JPN/Ishi-Im3/2015	2015	LC425410.1	Japan	7554
47	PSV/Porcine/JPN/Ishi-Im1/2015	2015	LC425409.1	Japan	7547
48	PSV/Porcine/JPN/HgYa2-4/2015	2015	LC425408.1	Japan	7475
49	PSV/Porcine/JPN/HgYa2-3/2015	2015	LC425407.1	Japan	7535
50	PSV/Porcine/JPN/HgYa2-2/2015	2015	LC425406.1	Japan	7515
51	PSV/Porcine/JPN/HgYa2-1/2015	2015	LC425405.1	Japan	7525
52	PSV/Porcine/JPN/HgTa2-2/2015	2015	LC425404.1	Japan	7525
53	PSV/Porcine/JPN/HkKa2-3/2015	2015	LC425403.1	Japan	7540
54	PSV/Porcine/JPN/HkKa2-2/2015	2015	LC425402.1	Japan	7555
55	PSV/Porcine/JPN/HgTa2-1/2015	2015	LC425401.1	Japan	7540
56	PSV/Porcine/JPN/Ishi-Miya3/2015	2015	LC425399.1	Japan	7508
57	PSV/Porcine/JPN/Ishi-Ka2/2015	2015	LC425398.1	Japan	7543
58	PSV/Porcine/JPN/Iba26-489S/2015	2015	LC425397.1	Japan	7531

59	PSV/Porcine/JPN/DeTk2-2/2015	2015	LC425396.1	Japan	7428
60	PSV/Porcine/JPN/HgOg2-5/2015	2015	LC425395.1	Japan	7480
61	PSV/Porcine/JPN/MoI2-2/2015	2015	LC425394.1	Japan	7425
62	PSV2020	2020	ON146286.1	China	7550
