# Supplementary material for: High Prevalence, Genetic Diversity, and Recombination of Porcine Sapelovirus in Pig Farms in Fujian, Southern China
Source: Viruses. 2023 Aug 17;15(8):1751. doi: 10.3390/v15081751 (PMC10458035; doi:10.3390/v15081751)
Supplement: Supplementary file 1 [file viruses-15-01751-s001.zip › Supplementary_Material-Figure/Supplementary_Material-Figure S1 Establishment of TaqMan-based real-time PCR for PSV.pdf]

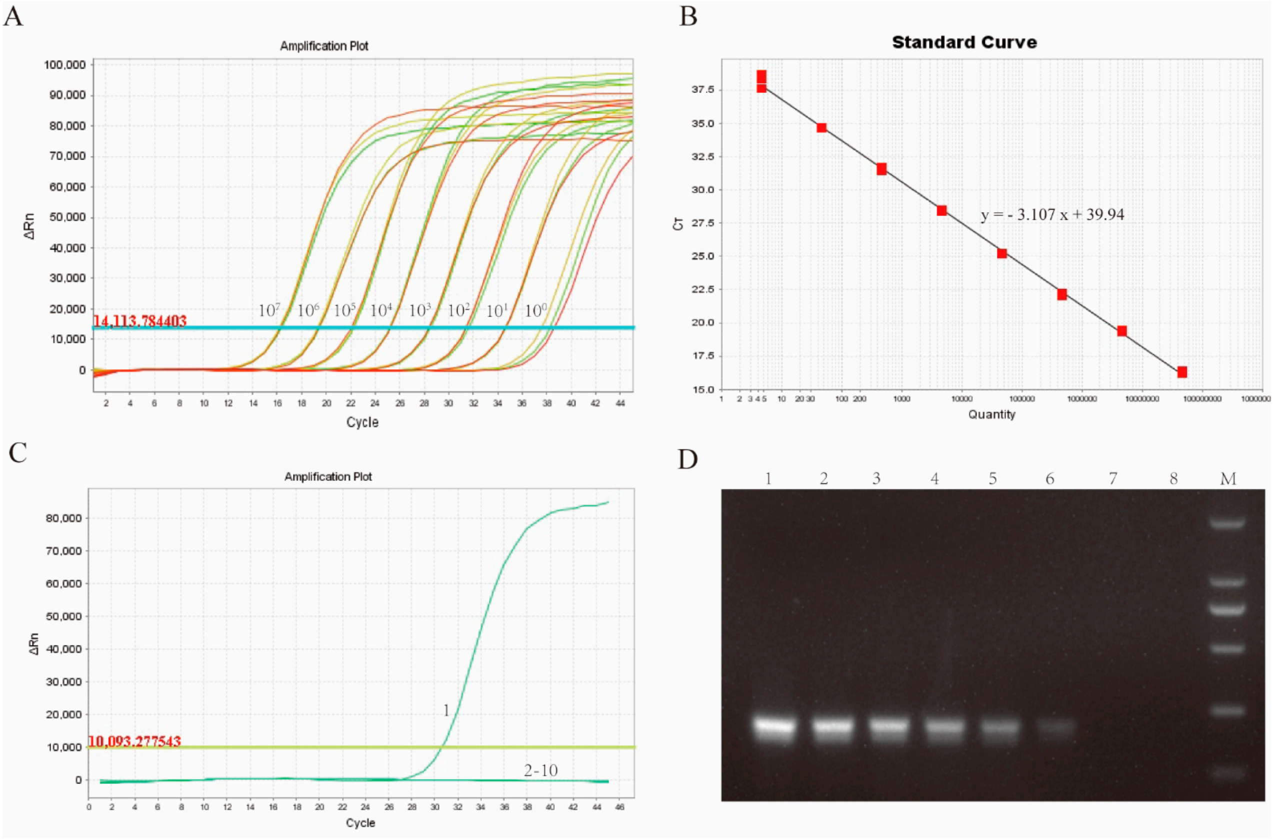

Supplementary Figure S1. Establishment of TaqMan-based real-time PCR for PSV.

(A) Amplification kinetics of real-time PCR. The assay was conducted using ten-fold serial dilutions of the plasmid with 4.67×10^7 ~ 4.67 copies/μL.
(B) Standard curve of real-time PCR.
(C) Specificity of the real-time PCR assay. 1: PSV; 2-10: CSFV, PRV, PRRSV, PEDV, TGEV, PoRV, PDCoV, PTV, and negative control.
(D) Sensitivity of conventional PCR for PSV detection. M: DL 2000 marker; 1-7 were 4.67×10^7 ~ 4.67×10^1 copies/μL plasmid concentration; 8: negative control.
