# Supplementary material for: High Prevalence, Genetic Diversity, and Recombination of Porcine Sapelovirus in Pig Farms in Fujian, Southern China
Source: Viruses. 2023 Aug 17;15(8):1751. doi: 10.3390/v15081751 (PMC10458035; doi:10.3390/v15081751)
Supplement: Supplementary file 1 [file viruses-15-01751-s001.zip › Supplementary_Material-Figure/Supplementary_Material-Figure S2 PSV whole genome PCR amplicon gel electrophoresis.pdf]

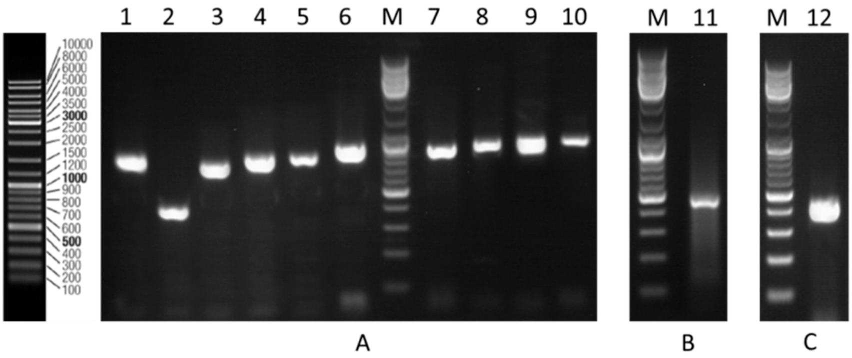

Supplementary Figure S2. PSV whole genome PCR amplicon gel electrophoresis.

(A) Internal region of PSV genome PCR amplification gel bands. (B) 3' RACE PCR amplification gel bands. (C) 5' RACE PCR amplification gel bands.
